# Should We Use Standard Survival Models or the Illness-Death Model for Interval-Censored Data to Investigate Risk Factors of Chronic Kidney Disease Progression?

**DOI:** 10.1371/journal.pone.0114839

**Published:** 2014-12-11

**Authors:** Julie Boucquemont, Marie Metzger, Christian Combe, Bénédicte Stengel, Karen Leffondre, on behalf of the NephroTest Study Group

**Affiliations:** 1 University of Bordeaux, ISPED, Centre INSERM U897 Epidemiology and Biostatistics, Bordeaux, France; 2 Inserm Unit 1018, CESP, Research Centre in Epidemiology and Population Health, Villejuif, France; 3 University Paris-Sud 11, UMRS 1018, Villejuif, France; 4 Centre Hospitalier Universitaire de Bordeaux, Service de Néphrologie Transplantation Dialyse, Bordeaux, France; 5 Unité INSERM 1026, Univ. Bordeaux, Bordeaux, France; Medical University of Graz, Austria

## Abstract

**Background:**

In studies investigating risk factors of chronic kidney disease (CKD) progression, one may be interested in estimating factors effects on both a fall of glomerular filtration rate (GFR) below a specific level (i.e., a CKD stage) and death. Such studies have to account for the fact that GFR is measured at intermittent visit only, which implies that progression to the stage of interest is unknown for patients who die before being observed at that stage. Our objective was to compare the results of an illness-death model that handles this uncertainty, with frequently used survival models.

**Methods:**

This study included 1,519 patients from the NephroTest cohort with CKD stages 1–4 at baseline (69% males, 59±15 years, median protein/creatinine ratio [PCR] 27.4 mg/mmol) and subsequent annual measures of GFR (follow-up time 4.3±2.7 years). Each model was used to estimate the effects of sex, age, PCR, and GFR at baseline on the hazards of progression to CKD stage 5 (GFR<15 mL/min/1.73 m^2^, n = 282 observed) and death (n = 168).

**Results:**

For progression to stage 5, there were only minor differences between results from the different models. The differences between results were higher for the hazard of death before or after progression. Our results also suggest that previous findings on the effect of age on end-stage renal disease are more likely due to a strong impact of age on death than to an effect on progression. The probabilities of progression were systematically under-estimated with the survival model as compared with the illness-death model.

**Conclusions:**

This study illustrates the advantages of the illness-death model for accurately estimating the effects of risk factors on the hazard of progression and death, and probabilities of progression. It avoids the need to choose arbitrary time-to-event and time-to-censoring, while accounting for both interval censoring and competition by death, using a single analytical model.

## Introduction

Chronic kidney disease (CKD) frequently follows a progressive path and is hardly reversible. CKD is classified into five stages of severity based on the level of glomerular filtration rate (GFR) and other markers of renal disease such as proteinuria and abnormal renal morphology [1], [2]. There are some interests to investigate factors associated with progression to some specific stage [3]. For example, Van Pottelbergh *et al.* [4] estimated the risk of a GFR fall below 15 mL/min/1.73 m^2^ in patients aged more than 50 years. LaMattina et *al.* [5] were interested in CKD progression from one stage to a more advanced stage in liver transplant recipients.

In all studies focusing on progression to a specific value of GFR, the time to progression is not known exactly and is said be “interval censored”. For instance, if the event of interest is a GFR fall below 15 mL/min/1.73 m^2^ (CKD stage 5) the time-to-event is interval censored between the last measure when GFR was above 15 mL/min/1.73 m^2^ and the first measure when it was below 15 mL/min/1.73 m^2^. Interval-censoring complicates survival analyses, in particular when some patients die before being observed at the stage of interest. Death, which can also be considered as an event of interest when investigating CKD progression, should be considered as a competing event since it precludes the observation of the stage of interest [6]. If the objective is to investigate the association between some risk factors and the hazard of progression to the stage of interest (i.e. estimating hazard ratios (HR)), competing risk by death can be accounted for by “censoring” at death in a cause-specific Cox model. Such an approach was for example used in LaMattina *et al.* [5]. However, censoring at death assumes that patients did not progress to the stage of interest in the time interval between the last measure of GFR and death. This is a strong assumption, especially if the time interval may be wide and if death is associated with renal function decline. Another option is to perform censoring at the last measure before death, rather than at death. However, censoring at a given time (last measure) because of what occurs at a later time (death) violates one of the major principles in survival analysis which consists in never conditioning on the future [7]. Censoring at the last visit before death has also been shown to produce biased effect estimates of factors that are associated with both the event of interest (e.g. progression to the specific stage of interest) and death [8]. The strength and the direction of bias depended on the strength and the direction of the effects of factors on the event of interest and on death, as well as on mortality rates. If the objective is to estimate the probability to progress to the stage of interest within a given period of time (i.e. the “cumulative incidence function” rather than the HR), then competing risks and interval censoring should also be accounted for.

The illness-death model for interval-censored data (IMID) avoids the need to choose a unique arbitrary censoring time by directly modeling the probability to progress to the stage of interest between the last measure of renal function and death. Such flexibility has been shown to produce much better estimates of both incidence rates of the event of interest (e.g. progression to the stage of interest) [9] and of the effects of risk factors that also affect death [8]. In addition, because the IMID allows the distinction between the hazard of death before and after the stage of interest, it gives a much more precise and complete picture of the course of the disease than a standard competing risks analysis that focuses only on death before the stage of interest. The IMID also allows estimation of the effects of risk factors on the hazards of all events (stage of interest and death) using a single model. Finally, the IMID also allows estimating the probability to experience the event of interest (i.e. progression to the stage of interest) within a given time window for patients with some given characteristics [10]. Despite these advantages, the IMID has never been used in the context of CKD [3]. It is thus unclear if it would conduct to different estimates of the effect of risk factors on the hazards of CKD progression and death, as well as of probabilities of progression, as compared to standard analyses. Yet, such comparison would be important to perform before recommending its use in CKD studies.

The main objective of this paper was thus to compare the results of the IMID with standard analyses for estimating the effects of factors on the hazard of progression to a specific CKD stage of interest and on death. To this aim, we compared the effect estimates of age, sex, proteinuria and GFR at baseline on the hazards of both CKD stage 5 and death, using data from the French NephroTest cohort study. The secondary objective was to illustrate the impact of modeling on the resulting estimates of probabilities of progression to CKD stage 5.

## Subjects and Methods

### Data source

The NephroTest study [11] is an ongoing prospective hospital-based cohort that began in January 2000. It includes patients with CKD stages 1 to 5 that are referred by nephrologists to any of three physiology departments for extensive work-ups including GFR measure (mGFR) by ^51^Cr-EDTA renal clearance and further followed-up [11], [12]. Included patients have to neither be on dialysis nor have received a kidney transplant. Pregnant women are excluded. All patients sign informed consent before inclusion in the cohort. The NephroTest study design was approved by the relevant ethics committee (Direction Générale pour la Recherche et l'Information, Comité Consultatif sur le Traitement de l'Information en matière de Recherche dans le domaine de la Santé MG/CP09.503) and adheres to the Declaration of Helsinki. As of December 31, 2010, a total of 1793 patients have been enrolled.

### Outcomes

CKD stage 5, defined by a mGFR fall below 15 mL/min/1.73 m^2^, was the primary event of interest. For patients who initiated renal replacement therapy before having been observed with a mGFR below 15 mL/min/1.73 m^2^, dialysis initiation or preemptive transplantation was considered as a first mGFR measure below 15 mL/min/1.73 m^2^. In the following, CKD stage 5 diagnosis therefore corresponds to the first actual mGFR measure below 15 mL/min/1.73 m^2^, dialysis, or preemptive transplantation, whichever came first. Dates of dialysis initiation and/or kidney transplantation were ascertained on December 31, 2010, either from medical records or through linkage with the national REIN registry of dialysis and transplantation [13].

Death was the secondary event of interest. The date of death or vital status on December 31, 2010, was ascertained by linkage with the National Identification Register of Private Individuals (RNIPP). For the few patients of Nephrotest who could not be retrieved in the REIN and RNIPP registry, administrative censoring was performed at their last follow-up visit before December 31, 2010.

### Statistical models

All models were proportional hazards models and provided regression coefficients estimates that all have hazard ratio (HR) interpretation. For all of them, we assumed Weibull distribution of the time-to-event because it generally fits well chronic disease incidence and mortality rates [8], [14]. Such parametric assumption facilitates the estimation of regression coefficients, especially when the number of events is relatively small.

**Figure 1:**
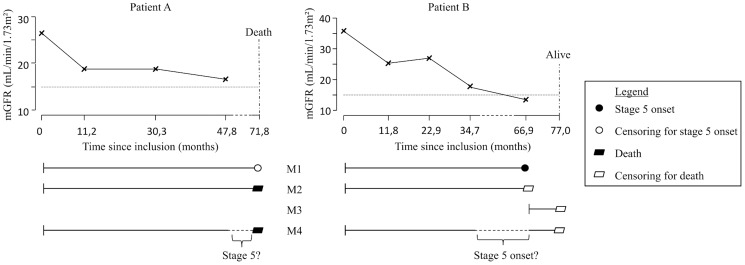
Times to event for chronic kidney disease (CKD) stage 5 and death used in models M1 to M4, for two patients: Patient A who died without prior CKD stage 5 diagnosis and Patient B who has been diagnosed with CKD stage 5.

The event of interest was CKD stage 5 in model M1. The time-to-event was the time elapsed from inclusion to CKD stage 5 diagnosis (patient B in Fig. 1), which was censored at the time to death or latest news on vital status for patients without CKD stage 5 diagnosis (Patient A in Fig. 1). Model M1 thus assumed that censored patients did not progress to CKD stage 5 in the time interval between the last mGFR measure and death or latest news.

The event of interest was death before CKD stage 5 diagnosis in model M2, and death after CKD stage 5 diagnosis but before kidney transplantation in model M3. In model M2, the time-to-event was the time to death before CKD stage 5 diagnosis (patient A in Fig. 1), which was censored at the time at CKD stage 5 diagnosis (patient B in Fig. 1). In model M3, the time-to-event was the time elapsed from CKD stage 5 diagnosis to death before kidney transplantation, which was censored at either kidney transplantation for those who received a transplant before death, or at the latest news on vital status for those who survived without kidney transplantation (patient B in Fig. 1). The few patients whose CKD stage 5 diagnosis corresponded to preemptive transplantation thus did not contribute to this analysis.

**Figure 2:**
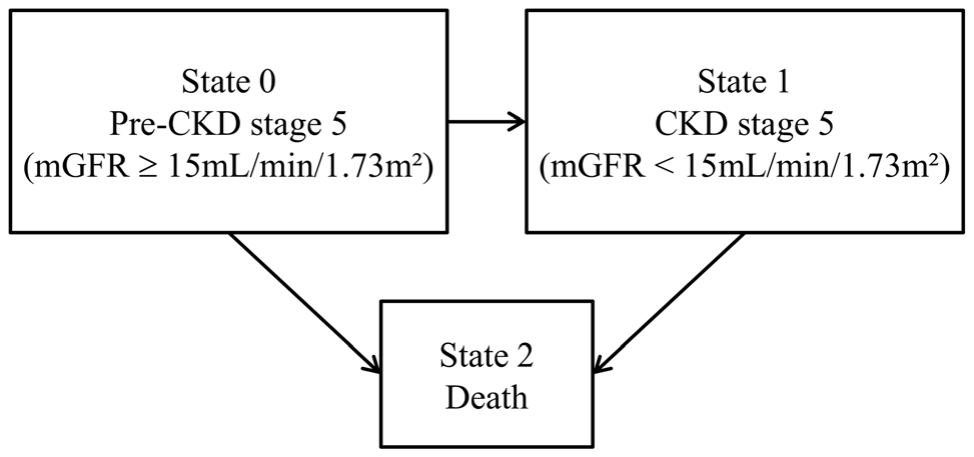
Graphical representation of the illness-death model (Model M4).

Both CKD stage 5 and death were the events of interest in the Weibull IMID M4. In this three-state model (Fig. 2), each patient was in state 0 at inclusion, i.e. in “pre-CKD stage 5” (mGFR ≥15 mL/min/1.73 m^2^), and may then have either: i) stayed in state 0 during all the follow-up (no transition), ii) progressed to state 1 (CKD stage 5) without dying thereafter (transition 01), iii) progressed to state 1 and then to state 2 (death) (transitions 01 and 12), iv) progressed directly to state 2 (transition 02). Model M4 which handled interval censoring of time to CKD stage 5, accounted for all these possibilities, including the possibility to have progressed through CKD stage 5 (state 1) in the time interval between a last mGFR measure above 15 mL/min/1.73 m^2^ (state 0) and death (state 2), as illustrated for patient A in Fig. 1. Note that, as in model M3, death was death before kidney transplantation, thus implying censoring at nonpreemptive kidney transplantation.

All models included sex, baseline age (in years), baseline log of protein/creatinine ratio (PCR in mg/mmol) and baseline mGFR (in mL/min/1.73 m^2^). Proportional hazard assumption on each transition (01, 02, and 12) was checked graphically for each variable as suggested in Leffondré *et al.* [8]. Log-linearity assumption for the effect of age, log PCR, and mGFR at inclusion was checked by comparing the results of the model using the continuous variable with the model using indicators of the quartiles of the variables [15].

HRs corresponding to each of these factors were derived from all models M1–M4. Because the IMID M4 has already been shown through simulations to systematically provide better HR estimates than standard models [8], we may assume that its HR estimates should be closer to true HRs. Large differences in HR estimates between the models should therefore indicate major biases of standard methods, and small differences minor biases. Probabilities of progression to CKD stage 5 within the next five years after inclusion were further derived for patients with some given characteristics (sex and values of age, log PCR, and mGFR at baseline) from the standard naïve model M1 and from the IMID M4 using the approach described in Touraine *et al.* [10]. We may expect differences between estimated probabilities from these two approaches since it is well known that in this context of prediction (i.e. when the aim is to estimate probabilities of event within a given period of time, rather than to estimate HR), the probabilities derived from naïve survival models censoring at the competing event may be largely biased, even when there is no interval censoring issue [16].

Estimated HRs from the Weibull survival models (M1 to M3) and the Weibull IMID model (M4) were obtained using the “eha” and “SmoothHazard” [17] R packages, respectively. Corresponding estimated probabilities of progression were also directly obtained from these packages.

## Results

### Patients' characteristics

Of the 1519 patients with CKD stages 1 to 4 at inclusion and without missing data on any risk factor, 282 were diagnosed with CKD stage 5 during the follow-up (Table 1). This included 139 patients who had at least one mGFR measure below 15 mL/min/1.73 m^2^, 128 patients who initiated dialysis without previous mGFR measure below 15 mL/min/1.73 m^2^, and 15 who received a preemptive transplant also without previous mGFR measure below 15 mL/min/1.73 m^2^. Among the 111 patients who died without CKD stage 5 diagnosis, the time interval between the last mGFR measure and death varied between 0.01 and 8.51 years. For 25% of these 111 patients, the time interval was longer than 3.5 years (Table 1), which may be potentially long enough to have progressed to CKD stage 5 before death.

**Table 1:** Characteristics of the study population (n = 1519, NephroTest cohort, 2000–2010, France).

Characteristics	n (%)	Mean (SD)	Percentiles		
			25^th^	50^th^	75^th^
Male	1040 (68.5)				
Age at inclusion (in years)	1519 (100.0)	58.9 (15.1)	48.9	60.7	71.2
mGFR at inclusion (in mL/min/1.73 m^2^)	1519 (100.0)	43.1 (18.4)	28.7	39.9	54.4
CKD stage at inclusion					
Stage 1 (mGFR ≥90)	28 (1.8)				
Stage 2 (60≤ mGFR <90)	250 (16.5)				
Stage 3a (45≤ mGFR <60)	332 (21.9)				
Stage 3b (30≤ mGFR <45)	479 (31.5)				
Stage 4 (15≤ mGFR <30)	430 (28.3)				
PCR at inclusion (in mg/mmol)	1519 (100.0)	84.4 (146.6)	10.4	27.4	90.0
Follow-up time (in years)	1519 (100.0)	4.3 (2.7)	2.2	3.8	6.4
Number of mGFR measures	1519 (100.0)	2.6 (2.0)	1.0	2.0	3.0
CKD stage 5 diagnosis	282 (18.6)				
mGFR <15 mL/min/1.73 m^2^	139 (9.2)				
Dialysis	128 (8.4)				
Preemptive transplantation	15 (1.0)				
Death	168 (11.1)				
Without CKD stage 5 diagnosis	111 (7.3)				
With CKD stage 5 diagnosis	57 (3.8)				
Time interval between last mGFR measure and death for patients died without CKD stage 5 diagnosis (years)	111 (7.3)	2.3 (2.0)	0.8	1.4	3.5

Abbreviations: SD, standard deviation; mGFR, measured glomerular filtration rate; CKD, chronic kidney disease; PCR, protein/creatinine ratio.

### Estimated effects of selected risk factors

For all selected risk factors, there were only minor differences between estimated effects on the hazard of progression to CKD stage 5 from the standard survival model M1 and the IMID M4. More specifically, sex and age were not statistically associated with progression to CKD stage 5 and the quantitative estimated effect of mGFR and log PCR at inclusion were similar with all the models. For example, an increase of one unit of log PCR at inclusion was associated with an estimated HR of 1.80 (95%CI: 1.62–2.00) in model M1 and 1.83 (95%CI: 1.63–2.06) in model M4-01 (Table 2).

**Table 2:** Association between sex, age, mGFR, and proteinuria at inclusion and hazard of progression to CKD stage 5 and death.

Variable	Model*	CKD stage 5		Death before CKD stage 5		Death after CKD stage 5	
		HR [95%CI]	p-value	HR [95%CI]	p-value	HR [95%CI]	p-value
Sex (female	M1	0.98 [0.76–1.26]	0.871				
vs. male)	M4-01	0.92 [0.71–1.19]	0.530				
	M2			0.58 [0.35–0.95]	0.032		
	M4-02			0.67 [0.36–1.24]	0.199		
	M3					0.94 [0.52–1.69]	0.831
	M4-12					0.71 [0.41–1.23]	0.223
Age (per 10	M1	0.96 [0.89–1.04]	0.344				
year	M4-01	1.03 [0.94–1.12]	0.535				
increase)	M2			2.17 [1.79–2.64]	<0.001		
	M4-02			2.19 [1.67–2.86]	<0.001		
	M3					1.77 [1.39–2.26]	<0.001
	M4-12					1.81 [1.43–2.29]	<0.001
mGFR (per	M1	0.34 [0.30–0.40]	<0.001				
10	M4-01	0.34 [0.30–0.40]	<0.001				
mL/min/1.73	M2			0.85 [0.74–0.97]	0.019		
m^2^ increase)	M4-02			0.91 [0.76–1.08]	0.269		
	M3					1.23 [0.89–1.71]	0.208
	M4-12					0.97 [0.70–1.36]	0.869
Log of PCR	M1	1.80 [1.62–2.00]	<0.001				
(per one unit	M4-01	1.83 [1.63–2.06]	<0.001				
increase)	M2			1.16 [0.98–1.36]	0.083		
	M4-02			1.10 [0.87–1.39]	0.423		
	M3					1.23 [0.97–1.54]	0.082
	M4-12					1.16 [0.91–1.47]	0.224

Abbreviations: CKD, chronic kidney disease; HR, hazard ratio; CI, confidence interval; mGFR, measured glomerular filtration rate; PCR, protein/creatinine ratio.

*M1, Weibull model imputing the time to progression to CKD stage 5 at the time at the first mGFR measure below 15 mL/min/1.73 m^2^, or censoring at death or latest news (n = 1519 patients contributed to the analysis); M2, Weibull model for death before CKD stage 5 diagnosis, censoring at the time at the first mGFR measure below 15 mL/min/1.73 m^2^ (n = 1519); M3, Weibull model for death after CKD stage 5 diagnosis (n = 245); M4, Weibull illness-death model accounting for interval censoring (n = 1519).

If interval censoring does not affect the results of standard models for death before CKD stage 5, we should expect that model M2 censoring at CKD stage 5 diagnosis gives results similar to model M4-02. This was the case for the effect of age: HR of 2.17 (95%CI: 1.79–2.64) for a 10-year increase in model M2 versus 2.19 (95%CI: 1.67–2.86) in model M4-02 (Table 2). The discrepancies between estimated HR from models M2 and M4-02 were larger for sex, mGFR and proteinuria at baseline. For sex, the HR comparing females to males was of 0.58 (95%CI: 0.35–0.95) in model M2 and 0.67 (95%CI: 0.36–1.24) in model M4-02. For mGFR at inclusion, the HR for a 10 mL/min/1.73 m^2^ increase was of 0.85 (95%CI: 0.74–0.97) in model M2 and 0.91 (95%CI: 0.76–1.08) in model M4-02. For proteinuria at inclusion, the HR for one unit increase in log PCR was of 1.16 (95%CI: 0.98–1.36) in model M2 and 1.10 (95%CI: 0.87–1.39) in model M4-02.

Similarly, if interval censoring does not affect the results of standard models for death after CKD stage 5, we should expect that the standard model M3 gives results similar to model M4-12. This was the case for the effect of age where the HR for a 10-year increase was of 1.77 (95%CI: 1.39–2.26) in model M3 and 1.81 (95%CI: 1.43–2.29) in model M4-12 (Table 2). The discrepancies between the HR estimates from models M3 and M4-12 tended to be larger for sex, mGFR, and proteinuria at inclusion but none of these HR was statistically significant.

### Impact of the modeling on resulting estimated probabilities of progression to stage 5

**Figure 3:**
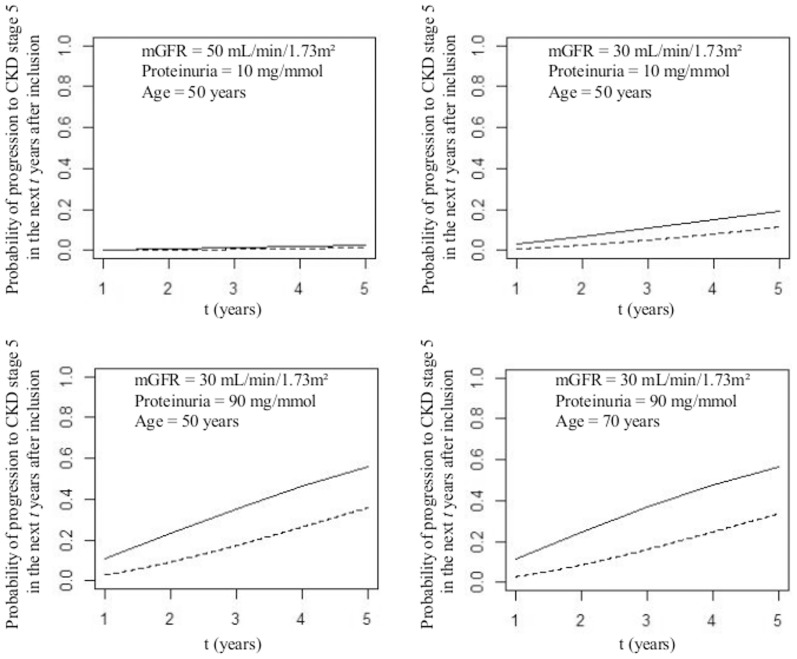
Probabilities of progression to CKD stage 5 in the next t years after inclusion into the cohort for a man with different ages, levels of mGFR and proteinuria at inclusion, estimated from the illness-death model for interval-censored data (IMID, model M4, solid line) and the standard survival model (model M1, dotted line).

The estimated probabilities to progress to CKD stage 5 were at each time higher with the IMID M4 than with the naïve survival Model M1 for the four the selected profiles of patients at inclusion (Fig. 3). For example, according to the IMID, a 50-year old man with a mGFR of 30 mL/min/1.73 m^2^ and a proteinuria of 90 mg/mmol at baseline had a probability of 0.56 to progress to stage 5 within the first five years after inclusion, while this probability was of only 0.36 with the naïve survival model M1.

## Discussion

While standard models and the IMID gave almost identical estimated effects of risk factors on the hazard of progression to CKD stage 5, the results tended to differ for death. In particular, we found a significant effect of mGFR at inclusion on the hazard of death before CKD stage 5 diagnosis in model M2 (p = 0.019), but not in the IMID M4-02 (p = 0.269). This may be due to a lack of power of the IMID, or to an over-estimation bias of the effect of baseline mGFR in model M2. Indeed, model M2 did not account for interval-censoring, i.e. did not account for the fact that patients who died without CKD stage 5 diagnosis might actually have progressed to stage 5 before dying. Accordingly, the effect of baseline mGFR on death before CKD stage 5 diagnosis in model M2 may partly reflect the strong effect of baseline mGFR on undocumented progression to CKD stage 5. Similar differences of results between M2 and M4-02 were found for the effect of baseline log PCR on the risk of death before progression (p = 0.083 versus p = 0.423).

For progression to CKD stage 5, the results were similar between standard model M1 and the IMID M4 likely because none of the investigated factors seemed to be significantly associated with both progression to CKD stage 5 and death, according to the IMID. Note that separating death from CKD stage 5 progression allowed us to clearly distinguish the effects of risk factors on each specific event. In particular, our results suggest that previous findings on the strong effect of age on end-stage renal disease [18] are more likely due to a strong impact of age on death than to an effect of age on progression to CKD stage 5. It would be of interest to replicate our comparison of models for factors that are likely to be strongly associated with both types of events since one might observe larger bias of standard methods for such risk factors, as shown in a previous simulation study [8]. Furthermore, it would also be of interest to compare the methods for other CKD stages. In this comparison study, we indeed focused on progression to CKD stage 5 only because this was the stage for which we had the largest number of events, and thus the strongest statistical power. Focusing on progression to CKD stage 5 indeed allowed us to increase the number of the primary event of interest by assuming that initiation of dialysis or preemptive transplantation could be considered as a mGFR measure below 15 mL/min/1.73 m^2^. This seems to be a reasonable assumption for most of the 128 patients who initiated dialysis without previous mGFR measure below 15 mL/min/1.73 m^2^, since only 10.5% of patients in France initiated dialysis with a GFR above 15 mL/min/1.73 m^2^ in 2011 [19]. The 15 patients who received a preemptive transplant without previous mGFR measure below 15 mL/min/1.73 m^2^ also likely had a mGFR below 15 mL/min/1.73 m^2^ at the time at transplant. Indeed, it has been recommended in France to propose enrolment of a patient on kidney transplantation waiting lists when GFR become below 20 mL/min/1.73 m^2^ and is likely to require renal replacement therapy within the next year or 18 months [20].

As expected, we found major differences in the probabilities of progression to CKD stage 5 derived from the naïve survival model M1 and from the IMID M4. In this context of prediction, the naïve survival model was not only biased because of interval censoring, but also because it did not appropriately account for competing risk by death. Indeed, while performing censoring at death in a survival model is sufficient to obtain estimates of HR if there is no interval censoring issue, it does not allow direct unbiased estimation of the probabilities of the event of interest [16]. Instead, these probabilities may be obtained from the Fine and Gray model that accurately accounts for competing risk [21]. However, the Fine and Gray model does not handle interval censoring. Thus, if the objective is to derive adjusted probabilities of progression to some specific CKD stage for some given patients' characteristics, one should rely on the IMID that accurately accounts for both competing risk and interval censoring.

In this study, we chose time-on-study as the time scale for all analyses. Because inclusion into the cohort did not correspond to any specific event in the patients' life course, apart from being referred to a participating center, we could have used age as the time scale [22]. However, because patients entered into the cohort at different ages, we would have to perform left-truncation at the age at inclusion [22], which would have implied too small risk sets for some ages given the wide range of age at inclusion (16.9–88.7 years). Moreover, while using age as the time axis would have allowed a perfect adjustment for age, it would have precluded the estimation of its effect.

Our paper focuses on the investigation of risk factors associated with progression to a specific stage of interest. If the interest is not on a specific CKD stage but rather on the overall quantitative trajectory of the renal function, then a linear mixed model with repeated measures of GFR as the outcome variable would be more appropriate [23]. The renal function trajectory can also be jointly modeled with hazards of death, dialysis, or transplantation to either account for potential informative dropout, or to perform dynamic prediction of these events.

In conclusion, even if the standard model offered similar estimates of the effect of selected risk factors on the hazard of progression to CKD stage 5, we believe that it is preferable to use *a priori* the IMID which is much more theoretically appropriate. There are several reasons for this preference. Firstly, these results cannot be generalized to all risk factors (especially to those would be associated with both progression and death), as well as to all cohorts (especially to cohorts with higher mortality rates). Secondly, we illustrated that we may have some differences between the models for the estimated effects on death before and after progression, which highlights the need to accounts for interval censoring even if one is only specifically interested in death before or after progression. Thirdly, the IMID simplifies greatly the analyses, by simultaneously estimating the effects of risk factors on all transitions (progression to the specific stage of interest, and to death before and after progression), thus providing a complete picture of all effects in a single run, while avoiding the need to choose arbitrary event and censoring times due to interval censoring. Finally, the IMID also allows accurate estimations of individual probabilities of progression for patients with given values of the risk factors. From a more general perspective, it could be of interest to use the IMID for investigating progression to any specific CKD stages, as well as progression to micro or macro-albuminuria, since the time to such events is also interval censored.
